# Supplementary material for: Data integration uncovers the metabolic bases of phenotypic variation in yeast
Source: PLoS Comput Biol. 2021 Jul 15;17(7):e1009157. doi: 10.1371/journal.pcbi.1009157 (PMC8315545; doi:10.1371/journal.pcbi.1009157)
Supplement: S1 File — Comparison of the posterior density distribution obtained by Hit and Run (HR) sampling with the Expectation Propagation (EP) algorithm. (PDF) [file pcbi.1009157.s001.pdf]

Supplementary Methods

Sampling the solution space

Let L denote the solution space of eq. 5 with constraints (eq. 6). Our aim is to sample random elements in the convex set L in order to characterize it by means of the posterior joint distribution of fluxes. This can be achieved using classical methods, such as the well-known **Hit and Run** algorithm (1). (2) turned to map the original problem of sampling the feasible solution space L into an inference problem of the joint distribution of metabolic fluxes, letting the linear and inequality constraints to be encoded within the likelihoods and prior probabilities, which via Bayes theorem provides a model for the posterior distribution density of the flux.

We compared the posterior density distribution obtained by Hit and Run (HR) sampling with the Expectation Propagation algorithm (EP). We ran HR with a burn-in length equal to 10^6 and a jump of 0.5, for a number of 10^6 to 10^7 iterations, and the EP algorithm with a high β parameter (Boltzmann inverse temperature parameter). S1 Fig hows the solution space sampled by HR (histograms) and the EP estimate (red curve). S2 Fig shows the Pearson correlation coefficients between variances and means estimated with EP and HR for different numbers of iterations. As can be seen, the Pearson correlation increases as the number of HR samples increases. Assuming that HR samples the true distribution of fluxes, means are well predicted by the EP algorithm, although variances are underestimated.

We further investigated whether the EP algorithm predicted well the variance-covariance matrix of the DynamoYeast fluxes. S3 Fig shows the relationship between 8 pairwise fluxes chosen at random, and the correlation ellipses (red curve) computed by the EP algorithm. As can be seen, the EP algorithm predicts well the variance-covariance matrix of fluxes, satisfying eq. 5, based on HR predictions.

References

[1] Meersche KVd, Soetaert K, Oevelen DV. xsample() : An *R* Function for Sampling Linear Inverse Problems. Journal of Statistical Software. 2009;30(Code Snippet 1). doi:10.18637/jss.v030.c01.

[2] Braunstein A, Muntoni AP, Pagnani A. An analytic approximation of the feasible space of metabolic networks. Nature Communications. 2017;8:14915. doi:10.1038/ncomms14915.
