# Supplementary figures and images for: Data integration uncovers the metabolic bases of phenotypic variation in yeast
Source: PLoS Comput Biol. 2021 Jul 15;17(7):e1009157. doi: 10.1371/journal.pcbi.1009157 (PMC8315545; doi:10.1371/journal.pcbi.1009157)

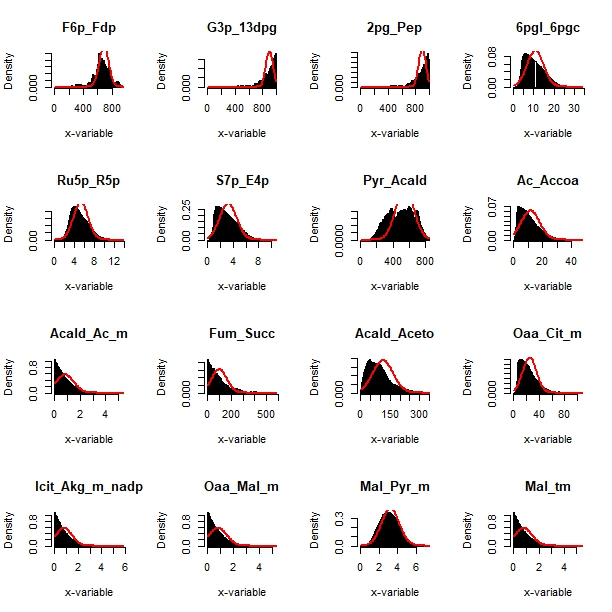

Supplement: S1 Fig — The histograms represent the HR result for T ∼ 107 sampling points. The red line is the result of the EP estimate. (TIF) [file pcbi.1009157.s002.tif]

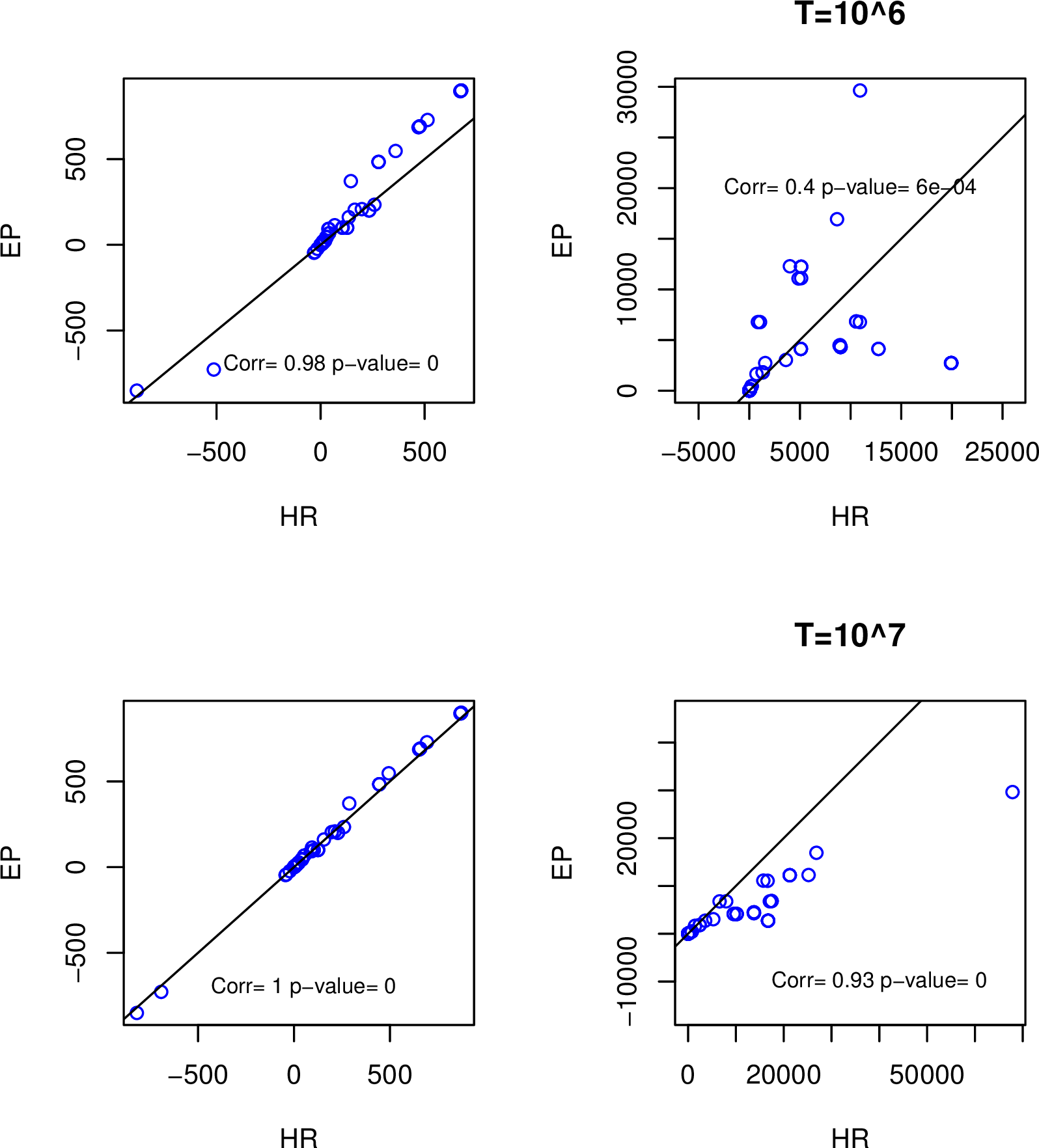

Supplement: S2 Fig — The plots on the left are scatter plots of the means and on the right variances of the approximated marginals computed via EP against the ones estimated via HR for an increasing number of explored configurations T, top T ∼ 106, bottom T ∼ 107. (TIF) [file pcbi.1009157.s003.tif]

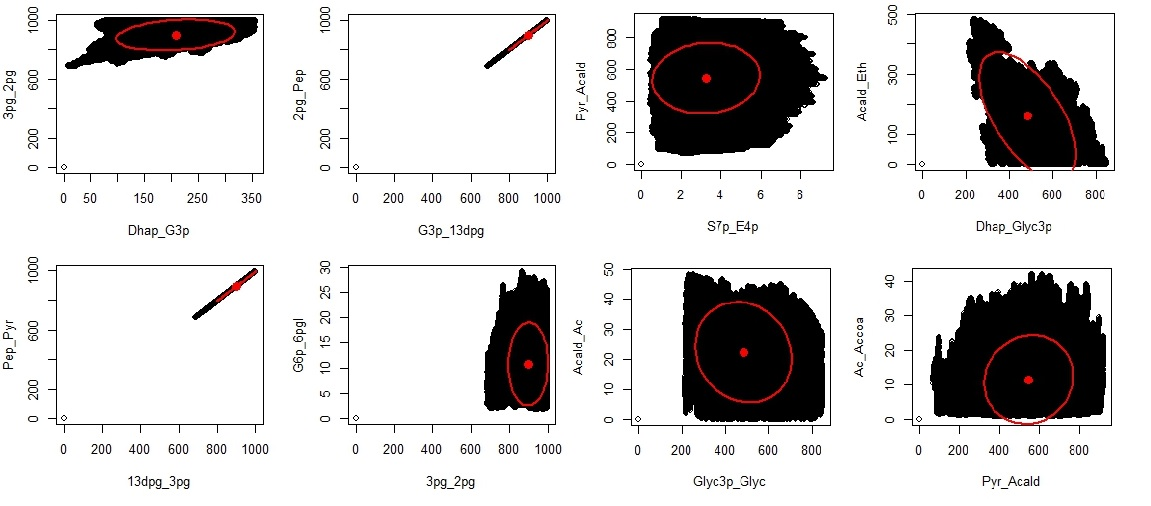

Supplement: S3 Fig — The plot shows the relationship between 8 pairwise fluxes. Correlation ellipses computed by the EP algorithm are drawn in red. Dot points represent the mean value of fluxes computed with EP. For HR samples, T ∼ 5 * 106. (TIF) [file pcbi.1009157.s004.tif]

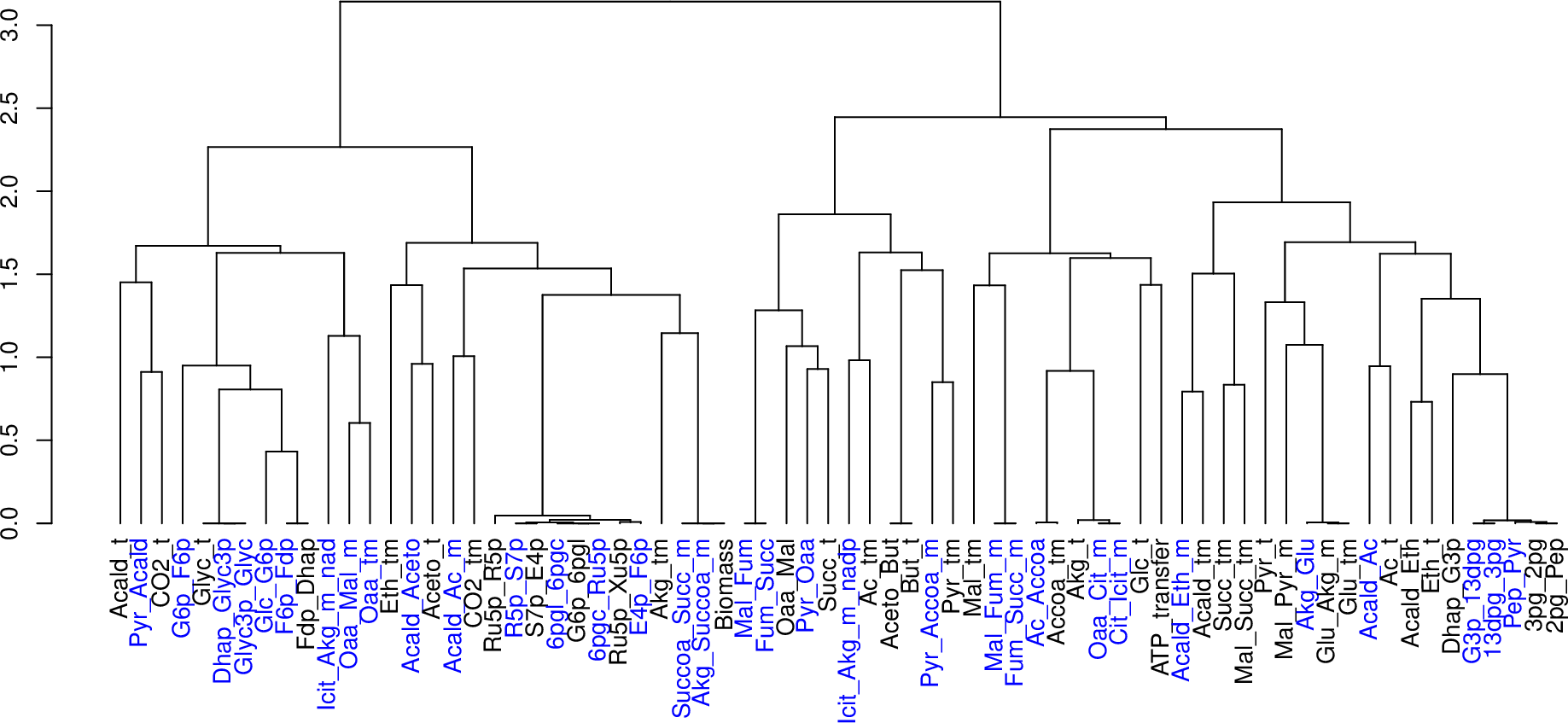

Supplement: S4 Fig — The null space of the stoichiometric matrix of the DynamoYeast model is spanned by the columns of the 70 reactions × 16 null-space matrix. Hierarchical clustering is applied using as a metric the angles between the 16 dimensional row vectors reactions. The reactions associated with enzymatic proteins quantified in this study are in blue, the other reactions present in the DynamoYeast model are in black. (TIF) [file pcbi.1009157.s005.tif]

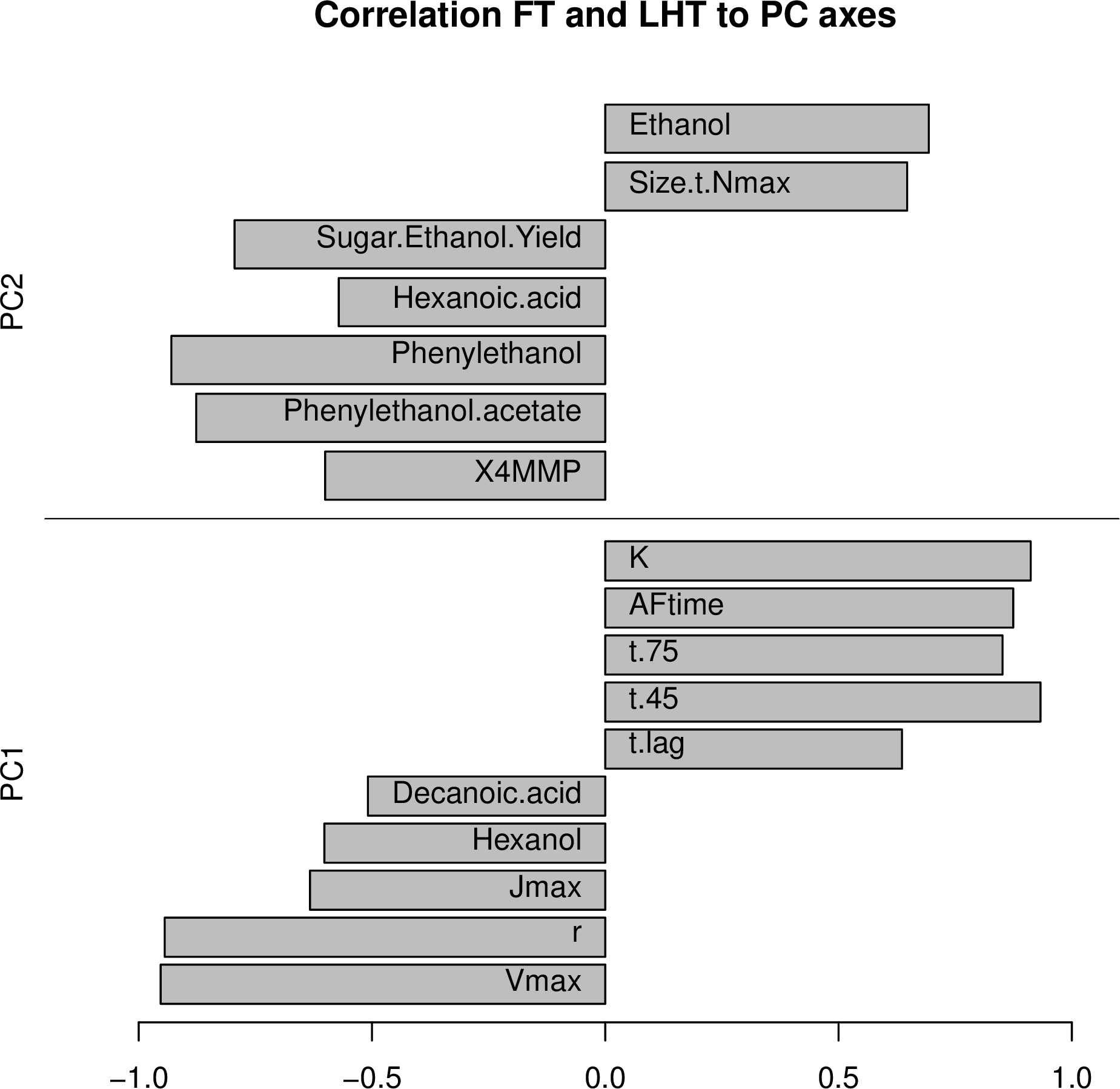

Supplement: S5 Fig — The figure shows traits for which the correlation was > 0.5 or < −0.5 (p-value < 0.05). The first axis is negatively correlated with growth rate (r), CO2 fluxes (Jmax and Vmax), Hexanol and Decanoic acid and positively correlated with carrying capacity (K) and fermentation times (AFtime, t-lag, t-75, t-45). The second axis is positively correlated with cell size (Size-t-Nmax) and Ethanol at the end of fermentation, and negatively correlated with aroma production at the end of fermentation and Sugar.Ethanol.Yield. (TIF) [file pcbi.1009157.s006.tif]

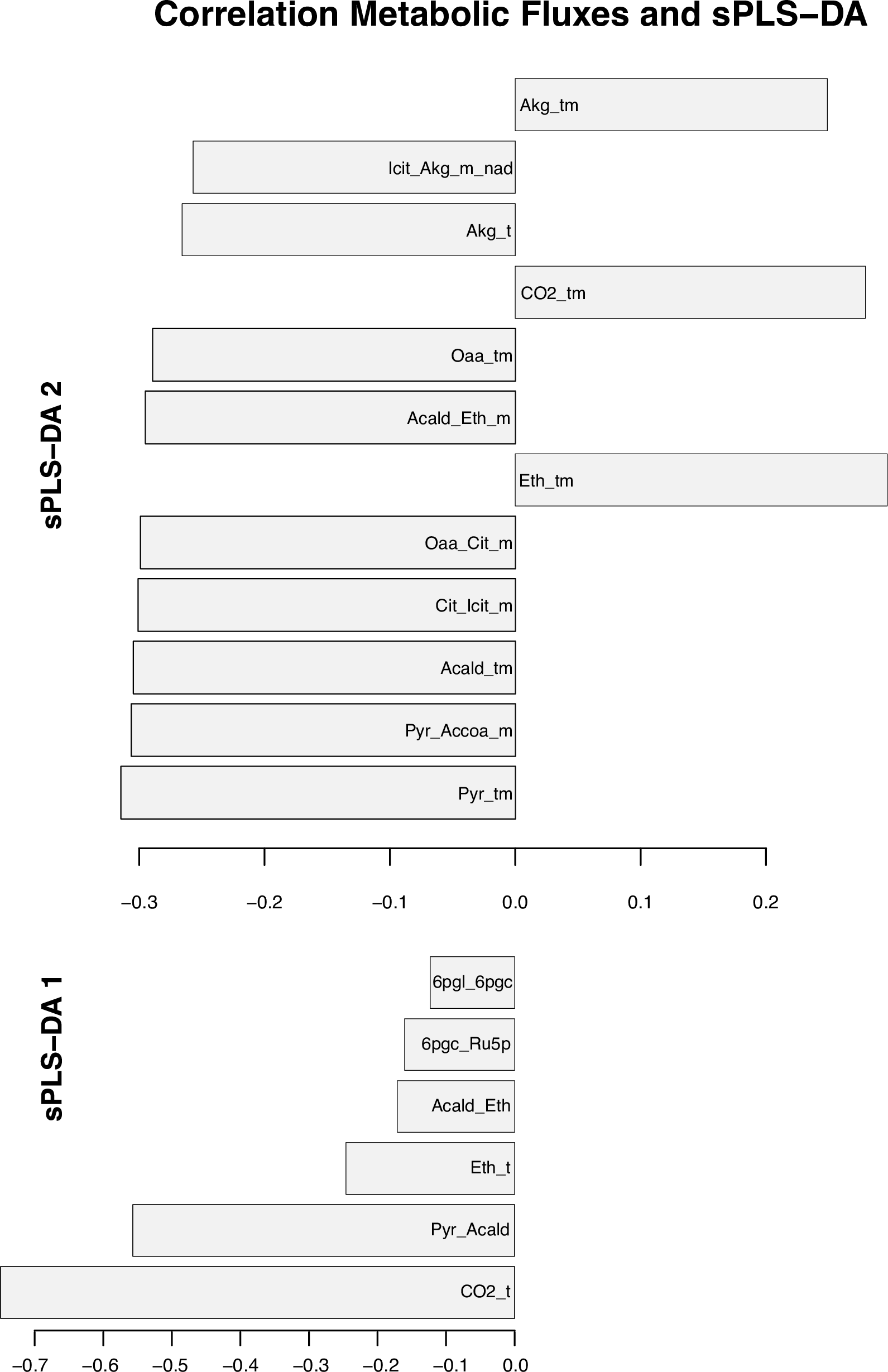

Supplement: S6 Fig — The CO2, pyruvate decarboxylase, ethanol, alcohol dehydrogenase, 6-phosphogluconolactonase and phosphogluconate dehydrogenase fluxes contributed to the first axis of the sPLS-DA, and were all negatively correlated with it. The second axis was negatively correlated with the mitochondrial acetyl-CoA formation, mitochondrial citrate synthase, mitochondrial aconitate hydratase, mitochondrial isocitrate dehydrogenase (NAD+) and mitochondrial transport fluxes of pyruvate, oxaloacetate and acetaldehyde fluxes, while positively correlated with the mitochondrial transport of 2-oxodicarboylate, ethanol and CO2 fluxes. (TIF) [file pcbi.1009157.s007.tif]

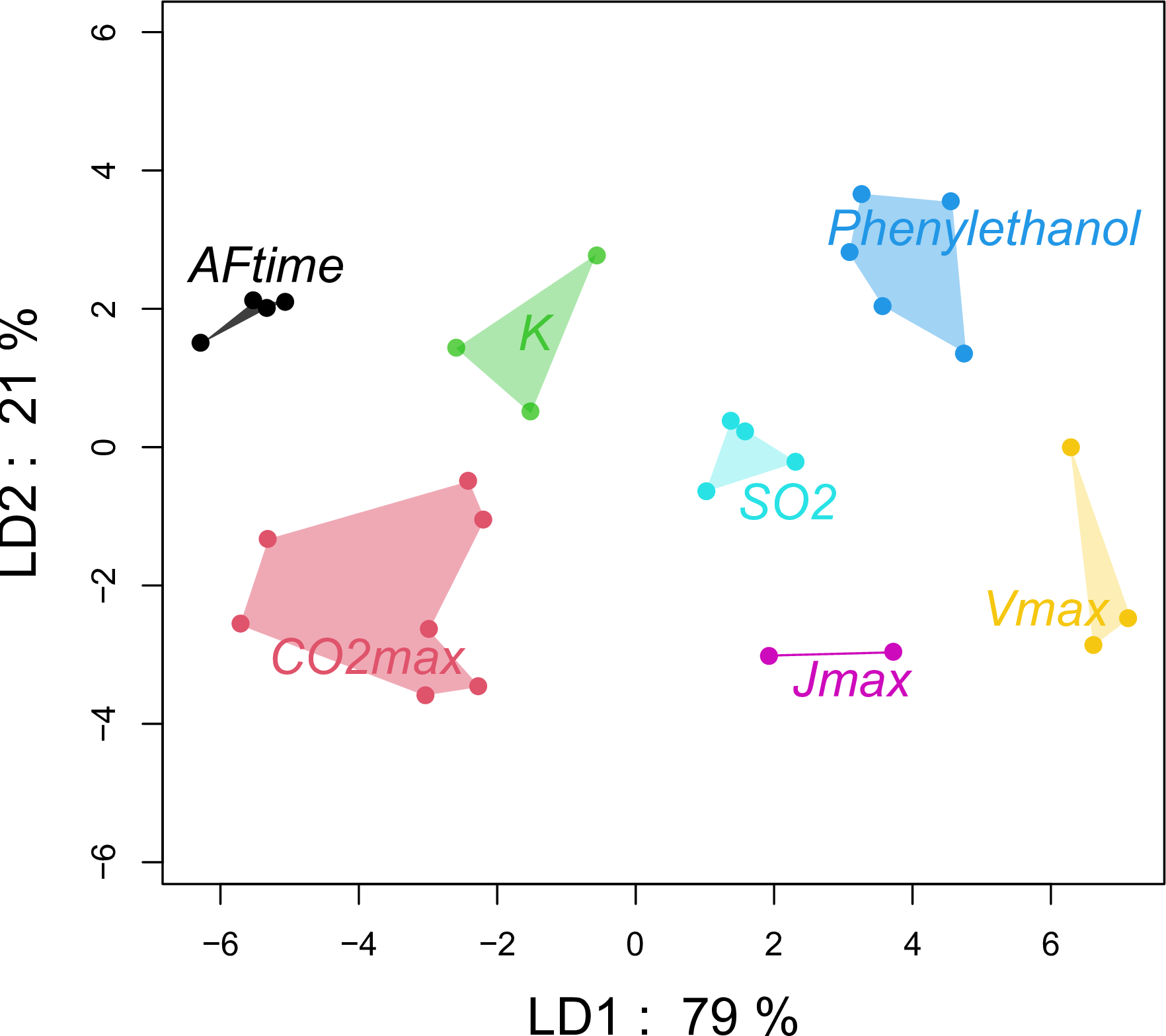

Supplement: S7 Fig — Projection of the 28 fermentation/life-history traits onto the first two axes of a Linear Discriminant Analysis of protein abundances. Each dot corresponds to one fermentation or life-history trait. (TIF) [file pcbi.1009157.s008.tif]
