# Supplementary material for: Data integration uncovers the metabolic bases of phenotypic variation in yeast
Source: PLoS Comput Biol. 2021 Jul 15;17(7):e1009157. doi: 10.1371/journal.pcbi.1009157 (PMC8315545; doi:10.1371/journal.pcbi.1009157)
Supplement: Attachment — Submitted filename: point-to-point_answer.pdf [file pcbi.1009157.s010.pdf]

Paris, May 11, 2021

To the Editors

Please find the revised version of our article “Data integration uncovers the metabolic bases of phenotypic variation in yeast”, by Marianyela Petrizzelli, Dominique de Vienne, Thibault Nidelet, Camille Noûs and Christine Dillmann (Ref: PCOMPBIOL-D-21-00127).

The issues raised by the Reviewers have all been addressed to improve and clarify various aspects of the manuscript. The changes have been marked in **blue** in the main text, and a point-by-point response is reported in the following text.

We hope that our article is now acceptable to be published in *PloS Computational Biology*.

Kind regards,

Marianyela Petrizzelli, on behalf of the authors

Reviewer 1 (Silvio Waschina):

Suggestions for manuscript improvement

Paragraphs at lines 41-58: It is not clear what the authors consider as high-throughput and what technique as low throughput. Also here, I am confident that many researchers would disagree that there are not current metabolomic approaches that can be considered high-throughput. E.g direct injection FTICR-MS provides you ten-thousands of masses and their intensities in less than a minute per sample. Thus, when discussing metabolomics in the context here, the authors should refrain from using the term low/high-throughput but instead clearly describe the potential and shortcoming of metabolomics techniques to understand phenotypic variation on metabolic flux level.

Lines 55-56: "Technical developments in mass spectrometry have boosted metabolomics by enabling the characterization of the metabolome, i.e. the complete set of metabolites in a cell.". Please rephrase and clarify this sentence. First, why mention only mass spectrometry and not mass spectroscopy? Especially since NMR was mentioned just two sentences before in the same paragraph. Second, there is to date no technique that is able to characterize all metabolites in a cell; each technique can measure only a specific range of metabolites (e.g. with respect to a specific mass range, polarity, hydrophobicity, etc.).

Lines 52-59. We thank the reviewer for this remark. Indeed, there is no technique able to characterize all metabolites in a cell. We removed the last part of the sentence. We mentioned mass spectrometry and not mass spectroscopy because it is well mass spectrometry that is used for metabolomics. (“Essentially, mass spectroscopy is the study of radiated energy and matter to determine their interaction, and it does not create results on its own. Spectrometry is the application of spectroscopy so that there are quantifiable results that can then be assessed” [https://verichek.net/spectroscopy-vs-spectrometry.html]).

Line 68-69. Why is this a specific population genetics view? Isn't it more a cell physiology/evolutionary view?

Line 70. Right. We deleted this non-relevant phrase.

Subsection 2.1 is very short. It begins by stating that the two algorithms (HT and EP) are compared; but it does not report any results from the comparison and only states that EP "gave a good approximation", without providing any quantitative results from the comparison. There's more detail in the appendix, but the reader would appreciate more details in the main text in order to understand the author's steps in this work.

Lines 138-148. We agree that this subsection 2.1 was too short and that the quantitative results were missing. So we added the required additional information.

A central notion in the manuscript is the distinction between "observable" versus "non observable" traits. Yet, the manuscript does not provide a clear definition for this distinction. For instance, are enzyme abundances "observable"; what about metabolite concentrations or reaction fluxes? Does non-observable mean that these traits are just difficult to measure?

We agree that the term “observable” is improper. We thus replaced “observable” traits with “integrated” traits or “high-level phenotypes”, terms that seem more suitable to us.

The use of the term "secondary metabolites" is somehow different than in most publications. I am aware that in the scientific community "secondary metabolites" is loosely defined, but pyruvate, succinate and acetate are usually considered metabolites of the central metabolism and not secondary. Thus, I would use a different term than secondary metabolites (e.g. lines 217, 234, 362) to prevent misunderstandings. Perhaps, in the context of the present work, a term like "minor fermentation products" would be more fitting?

We fully agree that pyruvate, succinate, acetate, etc. are not secondary metabolites. In the context of the present work we should rather distinguish between fermentation products and downstream metabolites, those that are produced in the downstream steps of the Krebs cycle. We modified the text accordingly.

Fig. 5: Word clouds are not a scientifically sound way of presenting quantitative data since visual differences might be misleading. Since the font size corresponds to the correlation of the respective fluxes in those groups with the LD1-axis, a better way to present this information would be a simple bar plot with the correlation value as bar height.

Fig 5. We thank the reviewer for this suggestion. Now we represent the functional enrichment results in a bar plot. Of note, we would like to underline that font size in the previous representation did not correspond to the correlation of the respective fluxes but on the proportion of proteins positively/negatively correlated to the LD1 axis belonging to a functional category divided by the proportion of proteins from the same category found in the MIPS database. We added this information in the caption for clarity.

Fig. 1: To make this figure accessible to readers who are less accustomed to common metabolite identifiers, it would be nice if wherever possible to write the full metabolite names in the network and provide abbreviation information in the figure legends for all remaining metabolite IDs.

Table S1. Because adding the names directly on the figure would make it too loaded, we added a supplementary table with the full metabolite names.

Figure 3D: Why is the x-axis scaled in a way that it shows ranges without data? If the scale is adjusted, the difference between groups might be more obvious from the visualization.

Fig 3D. Yes indeed. We modified the figure accordingly.

Reviewer #2:

The work by Petrizzelli er al. uses a constraint-based metabolic core model of S cerevisiae together with quantitative proteome data to predict metabolic flux distributions. These flux distributions parallel observations on the trait level and thus provide a rational and mechanistic interpretation.

In general, the work is interesting as it provides a data science approach to bridging disparate data sets. The presented work is sound, however, its main weak point is the lack of experimental validation. The authors aim to predict flux distributions in diverse yeast strains and confirm their validity indirectly by locking at phenotypic variation but lack validation at the flux level (at least for some strains). Given the many simplifications applied I think it is necessary to provide a direct experimental

validation at least for certain fluxes in selected strains to establish the feasibility of the suggested approach.

The goal of the work is to provide an original approach to bridge the gap between proteomic variation and high-level phenotypic variation, not to give estimates of real flux values. The strategy consisted in computing fluxes from the integration of data from different scales that reflect the dependency structure between observations. We applied this approach to a large experimental dataset as a proof of concept, and the results fully confirmed its validity. Besides, we here used a previously published dataset (from the HeterosYeast project) that did not include flux measurements other than the CO2 flux. Obtaining biologically sound results with limited information about flux values was among the objectives of the work, and this objective has been achieved.

Major

* The authors simulate growth on minimal glucose limited medium and compare it to experimental data on chemical complex medium. Please justify that assumption. In particular, why do the authors not expect any impact from amino acid metabolism or extracellular TCA supplements.

We thank the reviewer for this important point that we forgot to mention. Indeed the experimental data were obtained on yeasts grown on a complex medium close to enological conditions (Sauvignon blanc grape juice), while we simulated growth on minimal glucose medium. Despite this, we were able to obtain consistent results that show that the negative relationship between growth/fermentation traits and production traits is accounted for by a differential usage of the energy production pathway. This indicates the robustness of these processes with regard to the carbon source and gives more generality to the results.

In addition, this model has been previously used to study yeast growth on grape must. We added the following text and the corresponding reference in the Results section (lines 116-119): “In the DynamoYeast model, the only entry is glucose, and the model does not take into account the complexity of metabolism like the recycling of amino-acids or extracellular TCA supplements. However, it was shown to accurately predict growth on complex medium like grape must [26].”

We also added the following in the Discussion (lines 357-363): “Despite the fact that the DynamoYeast metabolic model is an oversimplified model of central carbon metabolism with glucose as the only external carbon source, we show that protein abundance variations were sufficient to capture quantitative changes in the orientation of central carbon metabolism that occurred between strains and between growing temperatures in our dataset. Even though our flux predictions may not be very accurate, we are confident that we captured the main patterns of flux variation. Predicting unobserved fluxes from observed protein abundances overall adds information about the functioning of the actual metabolic network.”

* The authors limit themselves to a core model of central carbon metabolism although for instance with yeast8 a highly curated metabolic model would be available too. It is even more surprising as the authors can therefore only use 33 protein abundance data of a much richer data set. This raises the concern that the observed correlation between the proteome and fluxome is a consequence of the very restricted degrees of freedom in the model. The authors should at least indicate the number of

independent fluxes and the overlap with their proteome. In addition, the authors should enlarge their model and verify that the observed correlation remains similar.

We added a subsection (lines 639-652) in the Material and Methods section that provides information about the size of the null space (Ker(S)=16) and its structuring into metabolic modules showing that the number of degrees of freedom is not too small regarding the size of the model. We also added sentences to indicate (section 2.2):

- that the correlations become more stable and less sensitive to the sampling of the reactions whenever the number of pseudo-observations exceeds the number of degrees of freedom (lines 167-169);

- that the simulations, along with the observation of the distributions of the observation between the metabolic modules, can be used to check the quality of the metabolic model coverage (lines 177-179).

We also added the following sentence in the Discussion (lines 382-384):

"The structure of the stoichiometry matrix allows defining metabolic modules that correspond to the main metabolic pathways [28]. Our simulations showed the importance of covering most metabolic modules with observations of protein abundances."

* the authors strictly use the GPR mapping, in particular they use min(P1,P2) for an AND association. In their data, how often do the authors see that P1 is upregulated, while P2 is downregulated? This could be a hint at post-translational regulation at those points and should be at least mentioned. What if you exclude such data ?

We added this information in section 4.1.4 of the Material and Methods (lines 542-548). All concerned pairwise correlations were either positive or null..

* L.95 "This approach …" I understand that this sentence refers to Fig 5 in particular. However, fig 5 is an LDA of Protein abundances. Thus, that conclusion could be drawn without use of the developed approach, couldn't it?

We thank the reviewer for this observation, which shows that this point was not clear in the manuscript.

Lines 94-95. We stated more clearly in the introduction that previous studies failed to find a clear link between proteomic data and integrated phenotypes.

Lines 665-680. We revised the Statistical analyses Method section (4.3) to better explain the approach, and we performed the same approach as in Fig 5 by discarding the flux level and directly studying the protein-trait relationship.

We revised the Results section 2.6 and added an additional Supplementary Figure (Fig S5). We show that neglecting the flux levels leads to a poorer discrimination between groups of traits. Besides, while this approach would allow us to find some proteins related with some trait groups, it would not allow us to connect this variation with changes in central carbon metabolism.

The end of the Introduction (lines 105-107) could not have been drawn without integrating the flux level. This was also specified in the Discussion (lines 405-406).

* L.148 “algorithm was efficient for” How was efficiency determined or measured? please define or reformulate

We wrote that the algorithm was actually efficient since the simulated fluxes were highly correlated to its initial values (see values in figure 2). We reformulated in the text (lines 170-179).

* L.294 “we were able to show that the metabolic flux level retains information” Please reformulate as you don#t know whether your predicted flux levels are correct.

Actually, we do not claim that our predicted flux levels are correct, we just write that they “retain information”. This statement seems to us valid, since introducing the predicted flux levels in our modeling allowed us to bridge the gap between proteomic data and integrated traits and to show that the negative relationship between growth/fermentation traits and production traits was accounted for by a differential usage of the energy production pathway.

* L.329 “Therefore, it is important that protein abundance observations cover the main features of the architecture …” This is a key point that I hinted above. However, the authors do not highlight how that can be achieved or what principle should govern that choice.

We thank the reviewer for this remark. We omitted this point in section 2.2. As explained above, we rewrote section 2.2 and added a new subsection in the Material and Methods section (lines 639-652) to better explain how we checked that the enzymatic proteins associated with the CBM were good predictors of metabolic fluxes by means of null space analysis and numerical simulations.

Minor

L.62, “The idea that a given set of environmental conditions will drive a cell to a steady state …” I think that is only true in the artificial setting of a chemostat but not true in any more realistic setting. Please reformulate.

Lines 63-64. Done. We rephrased into “The idea is to explore system's properties at a steady state, during which internal metabolites stay at a constant concentration while exchange fluxes are constant and correspond to a constant import/export rate.”

L.65 “the number of metabolites is much higher than the number of reactions.” It’s the other way round in a (genome-scale) metabolic model.

Indeed, we corrected it (line 66).

L..72 Please texpand your argument why [12] seems more promising a method than others. You say that fluxes should covary with enzyme abundance, essentially ignoring any post-translational regulation. Why should that be a realistic assumption?

We better explained our choice of the method [12] in the Introduction (lines 76-82).

Fig 1, CO_2 secretion is measured, so no need to give [0:1000] as ranges

We removed [0:1000] from Fig 1.

Fig 3, the two shades of blue are difficult to distinguish. please consider revising.

Yes indeed, we revised Fig 3.

Out of interest, since your model is small, could you have done an elementar flux mode/vector analysis and characterised the totality of the solution space explicitly rather than doing sampling?

Yes, we could have done it. However, the objective of the paper was to provide a proof of concept that could also be applied in a more realistic, higher dimension metabolic model.
